# Clinical Quantitative Evaluation of Tooth Wear: A 4-year Longitudinal Study

**DOI:** 10.3290/j.ohpd.a45075

**Published:** 2020-09-04

**Authors:** Bora Korkut, Dilak Tagtekin, Naci Murat, Funda Yanikoglu

**Affiliations:** a Assistant Professor, Marmara University, Faculty of Dentistry, Department of Restorative Dentistry, Istanbul, Turkey. Idea, hypothesis and experimental design, performed the overall experiments, wrote and proofread the manuscript.; b Professor, Marmara University, Faculty of Dentistry, Department of Restorative Dentistry, Istanbul, Turkey. Hypothesis and experimental design.; c Assistant Professor, Ondokuz Mayıs University, Engineering Faculty, Industrial Engineering, Samsun, Turkey. Consulted on and performed statistical evaluation.; d Professor, Istanbul Kent University, Faculty of Dentistry, Department of Restorative Dentistry, Istanbul, Turkey. Hypothesis, experimental design, proofread the manucript, contributed substantially to discussion.

**Keywords:** bruxism, colorimeter, fluorescence, occlusal splints, tooth wear, ultrasound

## Abstract

**Purpose:**

This study investigated the progression of incisal tooth wear clinically for 4-years, using various diagnostic methods. Effectiveness of occlusal splints (night guards) for patients with nocturnal bruxism was also evaluated.

**Materials and Methods:**

Forty maxillary incisors from 10 patients with nocturnal bruxism were selected. Group 1 (n=5) wore occlusal splints for 6 months, whereas group 2 (n=5) didn’t. Ultrasound, cast-model analysis (control), digital radiography, FluoreCam and colorimeter were used for measurements. Clinical progression of incisal wear monitored at baseline, 3, 6, 12, 24 and 48 months, respectively.

**Results:**

Ultrasound, cast-model analysis and FluoreCam readings gradually and statistically significantly decreased during the overall evaluation period for both groups (p<0.001). Regarding colorimeter, statistically significant differences in periodical measurements were observed from 24 months and 12 months, for group 1 and group 2, respectively (p<0.001). There were no statistically significant differences in readings at evaluation periods, between the groups, for ultrasound, digital radiography and cast-model analysis (p≥0.05); however, statistically significant differences were observed for colorimeter at 24 months (p=0.010) and 48 months (p<0.001), and for FluoreCam at 12, 24, 48 months (p<0.001). Annual decrease in mean crown length was determined as 20-30 µm for group 1 and 40-50 µm for group 2. The decreases in mean crown length were statistically significantly lower for group 1 compared to group 2, regarding the assessments for 1 year, 2 years and 4 years (p<0.001). Positive and good correlations were observed between ultrasound, cast-model analysis and FluoreCam measurements (p<0.001).

**Conclusions:**

Ultrasound, FluoreCam and colorimeter showed promising results for monitoring any change and progression of incisal tooth wear clinically. Ultrasound might be considered as a quantitative, reliable and repeatable method. Precision of the measurements varied among the diagnostic methods used. Occlusal splints may have a potential preventive effect for progressive tooth wear.

Tooth wear is irreversible loss of dental hard tissues due to a chemical or physical attack of non-bacterial origin.^26^ It is recognised as an increasing clinical problem among adolescents and even children, but also as a result of increased life expectancy and longer retention of natural teeth.^24^ The aetiology of tooth wear is often multifactorial, comprising effects of erosion, abrasion, and attrition.^26^ Individual susceptibility to tooth wear may be influenced by chemical, biological, behavioral, educational, cultural, dietary, occupational, and geographic factors.^12,36^

The prevalence of severe tooth wear with dentin exposure was found in 25% of a population, including 3% of 20-year-old and 15% of 70-year-old patients.^27,40^ Regarding permanent teeth, data on the physiological wear of occlusal enamel surfaces is rare,^24^ but has been reported to be around 15 μm/year for premolars and 29 μm/year for molars.^21^ A previous study found abrasion lesions on the palatal aspect of the incisal third of anterior teeth due to anterior guidance.^28^ Also, dentin exposure was commonly found on the incisal surfaces of anterior teeth with severe tooth surface loss.^2^

Defining and quantifying ‘pathological tooth wear’ is difficult clinically, but achievable in the laboratory. This term has been used to describe unacceptable levels of progressive wear.^24,33^ However, pathological tooth wear does not always mean that the wear condition is severe. Recent evidence showed that bruxism is generally associated with gastroesophageal reflux disease (GERD).^23,42^ The prevalence of bruxism ranges from 8%-31% within the general population; diurnal (daytime) bruxism affects 24% of the adult population and noctural bruxism 16%.^31^ In a 12-month period, longitudinal data from 70 patients with severe tooth wear demonstrated that, in most cases, the rate of progression was <15 μm; however, for some, especially those with reflux-related symptoms, it exceeded 100 μm in just a 6-month observation period.^32^

It is possible to prevent tooth wear at any stage of the condition only if a proper diagnostic procedure is performed.^41^ A systematic review indicated a lack of clinical studies focused on occlusal splints for patients with bruxism, considering tooth wear as the primary outcome, instead of pain.^31^ Splint mechanisms have been reported as: habit-breaking and/or muscle relaxation for patients with increased parafunctional habits, protection of dental tissues and jaws in terms of grinding and clenching, and repositioning of the condyles and jaws into centric relation.^10^ However, knowledge gaps remain regarding the effectiveness of occlusal splints, due to the lack of longitudinal clinical data.^31^ The certainty of evidence when comparing splints with no splints in patients with temporomandibular disorder was reported as ‘very low’ due to the high risk of bias, heterogeneity, and lack of precision in the estimates.^29,31^

Several indices have been introduced as a qualitative method to determine the extent and severity of tooth wear.^3,33,36,41^ Qualification by taking a thorough oral history and using validated questionnaires is the main aim of these indices. However, even with a detailed history and examination, the multifactorial origin of tooth wear often prevents a clear diagnosis of the aetiological factors, possibly making a definitive diagnosis unrealistic when dealing with patients suffering from long-established, recalcitrant chronic reflux disease^42^ or persistent bruxism,^31^ for example. Persistent bruxism, both diurnal and nocturnal, may greatly limit the prognosis of restorative materials.^24^ Moreover, a perpetually erosive oral environment may limit the longevity of partial coverage restorations. Therefore, the management may include wearing a protective occlusal splint at night (for nocturnal bruxism), myofeedback therapy (for diurnal bruxism), or medications such as proton-pump inhibitors (for GERD).^24^ Mechanical wear due to attrition or abrasion is enhanced by the initial demineralisation (softening) of dental hard tissue.^19^ As any tooth substance loss cannot be measured before the surface softens, tooth wear is often described as erosive tooth wear, although in all probability, abrasive processes contribute to tooth surface loss after chemical softening by erosion.^24,26^ Even with surface softening, the indices cannot detect changes at the micrometer level.^36^

Risk assessment, diagnosis of wear and progression monitoring are the keys to prevent or slow the irreversible effects of tooth wear. Priority should be given to the early, quantitative diagnosis of tooth wear and the decision for preventive interventions. Tooth wear can also be measured by quantitative techniques which tend to rely on objective physical measurements, such as groove depth, facet area, or crown height.^19^ The problem is, it takes years to detect the changes in the severity of enamel lesions, leading to lesions reaching into dentin. Thus, studies on interventions to control and inhibit dental tissue loss from wear have been limited to in vitro and in situ studies.^13^ The reasons have been the lack of quantitative, non-destructive techniques suitable for clinically, intra-orally evaluating the loss of dental hard tissue directly on the tooth surfaces.^16,19,24,36^ Previously, many devices and techniques were used for evaluating tooth wear. However, clinical assessment methods are still lacking.^16,19^

A systemic review of the literature by Joshi et al^16^ has identified the clinical diagnostic methods as: clinical examination, dental photography, indices, colorimetric procedures, quantitative light-induced fluorescence (QLF), optical coherence tomography (OCT), atomic absorbtion spectrophotometry (AAS), digital pH meter, micro-/nano-indentation and ultrasonication. Those authors also pointed out the difficulties in some methods for in vivo measurements, such as the need for polished and flattened surfaces for micro-/nano-indentation, limited intra oral accessibility for OCT, and the high cost of AAS and QLF.^16^ Ultrasound was presented as a non-destructive, quantitative, and effective method mainly in in vitro^4,19,25,35,38^ with only in a few in vivo^34^ trials. Joshi et al^16^ considered 0.3 mm as the lowest clinically detectable limit for ultrasound measurements, and reported that changes <0.3 mm might not be detected accurately. The ultrasonic system and probe type were considered effective factors in the sensitivity of the measurements. QLF was used to track wear on occlusal cusp tips previously in in vitro studies,^16^ and the grinding depth and fluorescence intensity of the teeth were strongly correlated. Dentin exposure was reported as increasing the fluorescence intensity of the teeth.^17,22^ Non-destructive longitudinal assessment success with problematic probe positioning for both QLF and ultrasound techniques were mentioned in the literature.^16^ However, both tools were considered as accurate methods for clinical early detection of tooth wear.^16^ Korkut et al^19^ reported that the accuracy of dental photography, digital radiography, and digital modelling methods were computer-software dependent and limited to 0.1 mm in their study. Operator experience, general camera and flashlight settings were also considered as important factors in dental photography, which might affect the detection sensitivity of early hard tissue loss.^16^ A colorimeter (Spectroshade, Dentsply Sirona; Konstanz, Germany) was also used for evaluating the correlation between light absorbance due to a formed colored complex and degree of lost material from the surface. It was reported to be a well-established method for measuring very short-term changes in erosive effects, but the lack of information on structural changes was also reported.^16^

The aim of this clinical longitudinal study was to quantitatively and non-destructively evaluate the progression of incisal tooth wear using various diagnostic methods as well as evaluate the reliability of the methods used. The null hypothesis was that the progression of tooth wear could not be monitored clinically.

## Materials and Methods

This study was approved by the Ethics Committee of Marmara University, Institute of Heath Sciences, approval date/No. was 24.12.2014/21. The committee consisted of the research and teaching staff of Medicine, Dentistry, Pharmacist, and Health Science faculties of Marmara University.

### Study Design and Inclusion/Exclusion Criteria

The study took place at a university clinic. Five male and five female adult patients with nocturnal bruxism were selected for this study. All selected patients were healthy, with no major systematic diseases, and an age range between 30 to 50 years, mean age 39 years. The presence of nocturnal bruxism was assessed according to The International Classification of Sleep Disorders (ICSD) criteria^39^ for sleep bruxism. The minimal selection criteria included symptoms of tooth-grinding or tooth-clenching during sleep and at least one the following: (a) abnormal tooth wear, (b) grinding sounds, (c) discomfort in the jaw muscles. The activity of jaw muscles during sleep was also considered as a criterion supporting the diagnosis. Patients who had other medical or mental disorders, such as sleep-related epilepsy and associated epileptic activity, which may cause abnormal movement during sleep, were excluded from the study.

After considering nocturnal bruxism, additional criteria for inclusion were: loss of a maximum of two teeth other than incisors and canines, lack of dental anomalies or partial-/full-ceramic restorations in anterior teeth, lack of malocclusion or crossbite and including worn incisal edges with minimal dentin exposure on maxillary incisors (occlusal/incisal wear severity scale,^15^ grade 2). Regarding these inclusion criteria for the patients, maxillary anterior central and lateral incisors (n = 4) of each patient were selected. The patients were divided into two groups. Twenty teeth in two female and three male patients (mean age 38.8 years) were included in the first group; patients were instructed to use night guards (stabilisation splints) for six months. Twenty teeth in three female and two male patients (mean age 39.2 years) were included in the second group and did not use night guards. All the patients used non-abrasive toothpaste (Pro-Expert Clinic Line Enamel Regeneration, Ipana, P&G; Cincinnati, OH, US) twice a day and random brand, non-abrasive, medium-bristle toothbrush.

The night guards were individually prepared on a cast model as hard occlusion splints for each patient in the first group. The splints were prepared using hard acrylic, generally 2 mm thick but reinforced to 4 mm in the anterior incisor section. The cervical borders of the splints were extended on the palatal side to 10–12 mm in length. Incisal borders in labial side were limited to the incisal third. The prepared splints were checked for full seating on all teeth, then the splints were occlusally adjusted for each patient to minimise abnormal muscle activity and produce neuromusculer balance.^29^ The occlusion of the splints was balanced in centric relation (CR), canine guidance was ensured bilaterally, and full contact of mandibular canines and incisors was adjusted to the splint surface during protrusive jaw movement using acrylic. All of the patients attended the monthly check-up appointments over the entire treatment period. The alterations of the occlusal contacts on the splints due to the repositioning of the mandible were checked and accordingly readjusted.

### Clinical Measurements

Five different non-destructive, quantitative diagnostic methods (ultrasound, digital radiography, cast model analysis, colorimeter, and FluoreCam [Therametric; Indianapolis, IN, USA]) were used to measure clinical progression of incisal tooth wear of maxillary incisors. Only the worn surfaces of incisal edges of the selected maxillary central and lateral incisors were measured at baseline, 3, 6, 12, 24, and 48 months.

Prior to baseline measurements, in order to create a stable reference point for cast analysis, a composite button (G-ænial AO2, GC; Tokyo, Japan) 2 mm in diameter was placed on the cervical third of each tooth to be measured. An etch-and-rinse adhesive system (Adper Single Bond 2, 3M Oral Care; St Paul, MN, USA) with 37% phosphoric acid (Condac 37, FGM; Joinville, SC, Brazil) and 40-s irradiance with an LED curing unit (Demetron Demi Ultra, Kerr; Orange, CA, USA) were used to create the buttons. Cast model analysis was performed on a cast of the maxillary arch of each patient and a new model was created for each follow-up using addition silicon. An industrial, digital screw-type micrometer (BMI 770150, Germany) with 0.5 mm tips was used for cast model analysis. Two tips of the micrometer were placed on worn incisal edges and the cervical edge of the composite buttons for each measurement. A single reading in µm was performed for each tooth at each follow-up for each group.

An industrial contact-type ultrasonic system (Novascope 4500, Harisonic, Staveley NDT; Kennewick, WA, USA) was used together with a micro-sensitive ultrasonic transducer (Harisonic, Staveley NDT) at 11 MHz frequency, with a tip diameter of 0.6 mm. Glycerine gel (Fisher Scientific; Hampton, NH, USA) was applied for better ultrasonic conduction. After calibration of the device using an aluminum thickness gauge, the teeth were isolated and the transducer was placed perpendicular to the incisal edge, the most mesial or distal edge of the worn surface of each tooth. This was done to individually measure the wear progression of enamel thickness. The transducer location for each tooth was recorded by taking a digital photograph to standardise positioning in subsequent measurements. During the ultrasound measurements, a staff member of the non-destructive detection technique (NDT) service accompanied the researcher. The potential alterations in angulation of the transducer were prevented by double checking from the digital screen of the ultrasonic device during the measurements. Since clinical tooth wear was monitored, the expected decrease in numerical value was very low and only the similar re-positioning of the probe would reveal the correct score on the screen. Obtaining the most similar ultrasonic wave diagram and numerical value count compared to the previous measurement on the screen was depended on the correct positioning and angulation of the transducer. Three readings were performed at each follow-up and the data was collected for both groups in micrometers.

Radiographic data of the teeth was also collected using phosphor plates (Vista Scan Mini Plus, Dürr Dental; Bietigheim-Bissingen, Germany). Periapical radiographs (0.08 s irradiation) were taken of the maxillary incisors. A parallel film holder (Endo Bite Senso Anterior, Kerr) was used to standardise repeated measurements. Integrated software (DBSWIN v 5.3.1, Dürr Dental) of the phosphor plates was used to determine the crown lengths of the teeth. A single reading using the ‘length’ tool of the software, between the worn incisal edges and labially most apical point of the crown, was taken for each tooth at each follow-up. The reference points of each tooth were saved as screenshots to standardise subsequent follow-ups. The data was collected for both groups in µm.

A clinical colorimeter (ShadeStar, Dentsply Sirona; Konstanz, Germany) was used to monitor the color changes of worn surfaces in terms of the light absorbtion/tissue darkness relation. An integrated VITA Classical Shade Guide scale (shade numbers 1 to 16) was used for the measurements. The colorimeter was calibrated, placed perpendicular to the worn incisal edges, and the shade number was deterimined after a single reading for each follow-up. Nominal scores from B1 to C4 were converted to numerical scores 1 to 16 for each shade in the VITA Classical Shade Guide. Thus, the digitised scores were used for comparisons between the follow-ups. The change in color was measured by subtracting a given digital score from the subsequent score. Thus, a greater difference indicated greater discoloration.

In addition, a light-induced fluorescence clinical device with a mechanism similar to that of QLF, FluoreCam, was used to monitoring mineral alterations on the worn dental surfaces. Measurements were performed in a dark room for more precise results. The teeth were isolated, disposable tips were placed perpendicular to and 1 cm from the worn incisal edges, and fluorescence images including the quantitative intensity values were collected for both groups at each follow-up. The intensity values were demonstrated as negative (-) units by FluoreCam and the magnitude of decrease indicated the amount of mineral loss from the worn surface.

### Evaluation and Statistical Analysis

The 4-year clinical progression of incisal tooth wear in the presence of nocturnal bruxism was observed in this study. The results at different time periods of the two patient groups were compared: group 1 (patients who used night guards for 6 months) and group 2 (patients who did not use night guards). Also the correlation between five different quantitative diagnostic methods (ultrasound, cast model analysis, digital radiography, colorimeter, and FluoreCam) was statistically evaluated.

Ultrasound, cast model analysis, and digital radiography techniques were used to quantify hard tissue loss on the worn incisal edges, but the reference points were different for each technique, resulting in different length measurements. Therefore, the difference values between the evaluation periods were taken into consideration for comparisons between the techniques using Pearson Correlation Coefficient statistics. The colorimeter quantified the darkening and FluoreCam quantified the mineral loss of the worn incisal surfaces. The measurement differences between the evaluation periods were also used for these two diagnostic techniques to evaluate the correlations. A single measurement was performed by a single operator for all diagnostic methods, with the exception of ultrasound. Two measurements by the same operator and one additional measurement by another experienced operator were performed using ultrasound in order to evaluate observer agreement. According to Cohen’s Kappa, perfect intra-observer (κ = 0.97 and 0.94) and inter-observer (κ = 0.92) agreements were obtained.

Statistical analyses were performed using IBM SPSS for Windows 23.0 (SPSS; Chicago, IL, USA). Intra- and inter-observer correlations for ultrasound measurements were determined using the ICC Test. Normality of data distribution was confirmed by the Shapiro-Wilk Test. Evaluations between the groups were made using the independent samples t-test. Evaluations within groups were made using repeated measures ANOVA. The correlation between the test methods was deterimed using Pearson’s correlation coefficient. The results of multiple comparisons were presented as mean ± standard deviation. The significance level was set at p < 0.05.

## Results

In this study, no statistically significant differences were observed between male and female patients regarding the data obtained by any of test methods used (p ≥ 0.05). Multiple comparisons in the groups and between the groups according to the diagnostic methods and evaluation periods are listed in Table 1.

**Table 1:** Multiple comparisons in and between groups according to diagnostic methods and evaluation periods

	Evaluation period	Group 1 (night guard use for 6 months)	Group 2 (no night guard use)	p
Ultrasound	0 months	2.272 ± 0.314^a^	2.261 ± 0.381^a^	0.919
	3 months	2.269 ± 0.314^b^	2.252 ± 0.381^b^	0.878
	6 months	2.267 ± 0.314^c^	2.244 ± 0.381^c^	0.836
	12 months	2.256 ± 0.314^d^	2.222 ± 0.381^d^	0.757
	24 months	2.231 ± 0.316^e^	2.178 ± 0.381^e^	0.634
	48 months	2.169 ± 0.316^f^	2.087 ± 0.383^f^	0.462
	p	<0.001	<0.001	
Digital radiography	0 months	10.58 ± 1.079	10.715 ± 0.78^a^	0.653
	3 months	10.58 ± 1.079	10.715 ± 0.78^a^	0.653
	6 months	10.58 ± 1.079	10.715 ± 0.78^a^	0.653
	12 months	10.58 ± 1.079	10.715 ± 0.78^a^	0.653
	24 months	10.58 ± 1.079	10.615 ± 0.78^b^	0.907
	48 months	10.48 ± 1.079	10.515 ± 0.78^b^	0.907
	p	1.000	<0.001	
Cast model analysis	0 months	7.729 ± 1.167^a^	7.796 ± 0.989^a^	0.845
	3 months	7.725 ± 1.167^b^	7.785 ± 0.990^b^	0.861
	6 months	7.721 ± 1.168^c^	7.775 ± 0.990^c^	0.875
	12 months	7.709 ± 1.167^d^	7.753 ± 0.991^d^	0.899
	24 months	7.686 ± 1.167^e^	7.705 ± 0.991^e^	0.956
	48 months	7.626 ± 1.170^f^	7.610 ± 0.990^f^	0.963
	p	<0.001	<0.001	
Colorimeter	0 months	6.650 ± 2.300^a^	6.800 ± 2.526^a^	0.845
	3 months	6.650 ± 2.300^a^	6.800 ± 2.526^a^	0.845
	6 months	6.650 ± 2.300^a^	6.800 ± 2.526^a^	0.845
	12 months	6.850 ± 2.254^a^	8.100 ± 2.269^b^	0.089
	24 months	7.600 ± 2.326^b^	9.550 ± 2.212^c^	0.010
	48 months	8.900 ± 2.245^c^	11.95 ± 1.638^d^	<0.001
	p	<0.001	<0.001	
FluoreCam	0 months	-9.576 ± 0.898^a^	-8.477 ± 1.53^a^	0.009
	3 months	-10.522 ± 0.975^b^	-10.86 ± 1.58^b^	0.421
	6 months	-11.633 ± 0.962^c^	-11.689 ± 6.066^ab^	0.968
	12 months	-13.58 ± 1.152^d^	-16.775 ± 1.504^c^	<0.001
	24 months	-17.39 ± 1.428^e^	-22.315 ± 1.752^d^	<0.001
	48 months	-23.024 ± 1.841^f^	-29.754 ± 1.873^e^	<0.001
	p	<0.001	<0.001	

Same superscript letters indicate no statistically significant differences between groups.

There was no statistically significant difference between the groups at baseline and each evaluation period for ultrasound, digital radiography, and cast model analysis measurements (p ≥ 0.05). Statistically significant differences were observed between the groups for colorimeter measurements at 24 months (p = 0.010) and 48 months (p < 0.001). Only FluoreCam measurements were statistically significantly different at baseline, initially revealing different amounts of minerals between the groups. There were also statistically significant differences for FluoreCam readings between the groups at 12, 24, and 48 months (p < 0.001).

**Fig 1:**
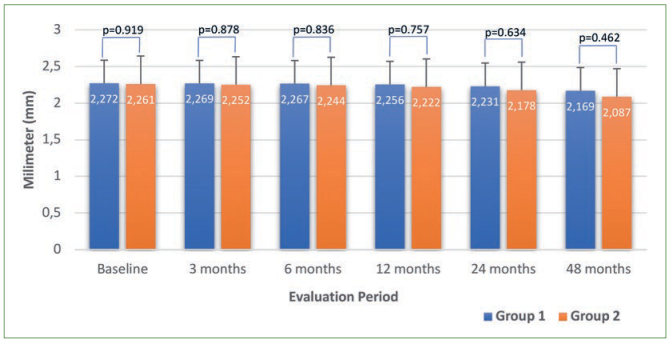
Diagram of ultrasound measurements according to the groups and evaluation periods (mm).

Ultrasound readings gradually decreased during the overall evaluation period and the measurements at each evaluation period were statistically significantly different for both groups (p < 0.001) (Fig 1).

**Fig 2:**
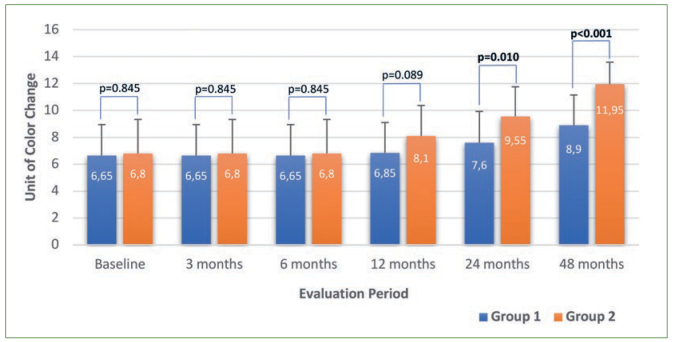
Diagram of colorimeter measurements according to the groups and evaluation periods (unit of color change).

**Fig 3:**
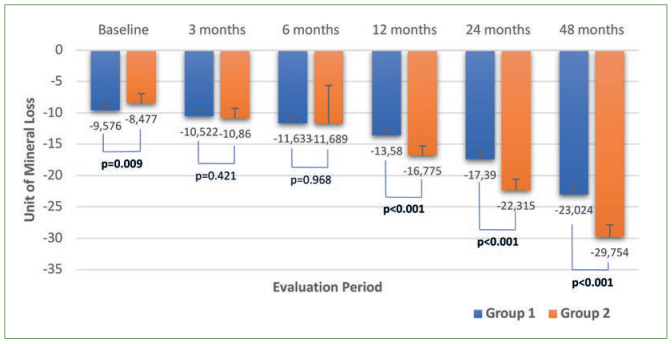
Diagram of FluoreCam measurements according to the groups and evaluation periods (unit of mineral loss).

Regarding digital radiography readings, no statistically significant difference was found for group 1 during the overall evaluation period, whereas the measurements at 24 and 48 months were statistically significantly different (p < 0.001) for group 2. Similar to the ultrasound results, cast model analysis readings gradually decreased during the evaluation period and measurements between each period were statistically significantly different for both groups (p < 0.001). Regarding the colorimeter, the measurements at 24 months and 48 months were statistically significantly different in terms of darkening in color for group 1. Also the measurements at 12, 24, and 48 months were statistically significantly different for group 2 (Fig 2). FluoreCam readings also gradually decreased during the evaluation period for both groups, revealing the increasing amount of mineral loss. The measurements at each evaluation period were statistically significantly different for groups 1 and 2 (except readings at 6 months for group 2) (p < 0.001) (Fig 3).

The differences in measurements between the evaluation periods were specified as 3 months-(minus) baseline (T1), 6 months-3 months (T2), 12 months-6 months (T3), 24 months-12 months (T4), and 48 months-24 months (T5). The correlations between the diagnostic methods for T1-T5 are given in Table 2. There was a positive, good correlation between ultrasound and FluoreCam measurements in terms of the differences in measurements for the overall evaluation period (T1-T5) (p < 0.001). Similarly, the correlation between ultrasound and cast model analysis was determined to be positive and very good for the overall evaluation period (p < 0.001). There was no correlation between ultrasound and digital radiography at all, except at T4, due to no detectable changes in measurements at baseline, 3 months, 6 months, and 12 months. Also no correlation was found between ultrasound and colorimeter at T1 and T2, as no detectable changes were recorded for measurements at baseline, 3 months and 6 months. In contrast, a positive and good correlation was found for T3, T4, and T5 (p < 0.001).

**Table 2:** Correlations between the diagnostic techniques regarding the differences in measurements between the evaluation periods (T1-T4)

Correlation between ultrasound and FluoreCam				Correlation between ultrasound and colorimeter		
	r	p			r	p
T1	0.667	<0.001		T1	---	---
T2	0.587	<0.001		T2	---	---
T3	0.735	<0.001		T3	0.599	<0.001
T4	0.787	<0.001		T4	0.366	<0.001
T5	0.749	<0.001		T5	0.629	<0.001
						
Correlation between ultrasound and cast model analysis				Correlation between ultrasound and digital radiography		
	r	p			r	p
T1	0.753	<0.001		T1	---	<0.001
T2	0.631	<0.001		T2	---	<0.001
T3	0.83	<0.001		T3	---	<0.001
T4	0.767	<0.001		T4	0.867	<0.001
T5	0.729	<0.001		T5	---	<0.001

*T1: 3 months-baseline; T2: 6 months-3 months; T3: 12 months-6 months; T4: 24 months-12 months; T5: 48 months-24 months.

Regarding groups 1 and 2, the decreases in the mean crown length for maxillary incisors were 20 µm and 43 µm (after 1 year), 43 µm and 91 µm (after 2 years), and 103 µm and 186 µm (after 4 years), respectively. According to the independent samples t-test, the differences between the groups were statistically significant at the 1-year (p < 0.001), 2-year (p < 0.001) and 4-year (p < 0.001) follow-ups (Table 3).

**Table 3:** Comparisons between the groups regarding decreases in mean crown length

	Group 1	Group 2	p
Baseline – 1 year	0.020 ± 0.0023	0.043 ± 0.0039	<0.001
Baseline – 2 years	0.043 ± 0.0039	0.091 ± 0.0053	<0.001
Baseline – 4 years	0.103 ± 0.0049	0.186 ± 0.0071	<0.001

## Discussion

The null hypothesis was rejected, as the progression of tooth wear could be monitored clinically. Ultrasound, cast model analysis, and FluoreCam detected the progression of incisal tooth wear for the overall evaluation period. The colorimeter detected the changes in color with limitations, and digital radiography was almost unable to detect the progression of tooth wear under the conditions of this study. Most of the studies reported that males showed more advanced tooth wear than females,^24,27^ whereas other authors did not find gender to be an influencing factor.^30^ In the present study, gender did not influence incisal tooth wear (p ≥ 0.05).

Because the data in our study were quantitative, not binary, sensitivity, spesificity and accuracy could not be analysed. Repeatability of ultrasound was analysed using Cohen’s Kappa Statistics. Also correlations between the test methods were evaluated to obtain reliability using Pearson’s Correlation Coefficient.

### Assessment of the Ultrasound Measurements

Ultrasound has been used to detect longitudinal measurement of progressive enamel loss.^8,14,16^ However, a paucity of longitudinal clinical trials still exists.^13^ Especially the repeatability and reliability have been a concern because of poor probe-tip positioning.^16^ For instance, Louwerse et al^25^ investigated ultrasound for measurement of enamel thickness in an in vitro study and reported poor repeatability due to probe positioning variations. Furthermore, enamel thickness <0.33 mm was determined to be undetectable. In contrast, many studies have mentioned ultrasound as a promising method for measuring enamel thickness. Tagtekin et al^38^ and Bozkurt et al^4^ reported ultrasound as a feasible and reliable method for measuring the enamel thickness on occlusal cusp tips in vitro. Sindi et al^35^ observed high accuracy for enamel thickness measurements with ultrasound, which also agreed well with histological findings. Those authors reported the potential for monitoring the progressive loss of enamel thickness on erosive tooth surfaces. Tagtekin et al^37^ reported accuracy and high repeatability for ultrasound measurements in detecting approximal caries lesions and also a positive correlation with DIAGNOdent measurements. Huysmans and Thijssen^14^ used ultrasound for in vitro longitudinal measurement of progressive enamel loss. They obtained a very good correlation between ultrasound and microscopic measurements. Ultrasound was reported as a feasible method for measuring enamel thickness with good interobserver agreement for cervical, mid-buccal, incisal, and palatal site measurements. The limitation posed by the thickness of the aluminum calibration block was mentioned as the reason for 0.5 mm sensitivity. Hughes et al^13^ measured the enamel thickness of progressively abraded tooth samples and reported that ultrasound was capable of measuring enamel thickness with an accuracy of 10% of total enamel thickness. However, they also mentioned the lack of clinical tools available to measure enamel thickness. Korkut et al^19^ also used ultrasound in vitro to evaluate the effectiveness and accuracy of measuring progressive wear on incisal surfaces, finding that ultrasound and digital micrometer measurements were positively and significantly correlated, and that ultrasound successfully monitored progressive incisal tooth wear down to < 50 µm. In a clinical study, Sindi et al^34^ investigated the reproducibility of ultrasound for measuring enamel thickness in maxillary central incisors. Those authors reported ultrasound measurements to be reliable and reproducible, with good inter-observer agreement for cervical and mid-buccal sites, but low reproducibility for the incisal site. Recently, diagnostic performance of ultrasound was investigated by Yanikoglu et al^43^ for flat surface caries lesions with different depths, and by Kim et al^17^ for early caries (white-spot) lesions. According to their results, ultrasound could quantify both the flat surface caries and white spot lesions.

In the present longitudinal clinical follow-up study, ultrasound readings gradually statistically significantly decreased for all the evaluation periods (p < 0.001) (Fig 1). A possible interpretation is that ultrasound may be able to clinically monitor progressive incisal tooth wear of less than 10 µm on maxillary incisors. This was a better result compared to the 50 µm in a previous study by Korkut et al.^19^ The positive, statistically significant correlation obtained between ultrasound and cast-model readings (T1-T5) (Table 2) points to clinical reliability of ultrasound, which was consistent with results by Hughes et al,^13^ Korkut et al,^19^ Bozkurt et al,^4^ and Sindi et al.^35^ Ultrasound for measuring worn incisal surfaces may also be a repeatable technique, given the almost perfect intra- (κ = 0.97 and 0.94) and inter-observer (κ = 0.92) agreement in the present study. Although this result seems to contradict that of the clinical study by Sindi et al,^34^ their ultrasonic transducer was located labially, not incisally, at the incisal site. Thus, the ultrasonic transducer was located on a smaller worn incisal surface in our study, probably reducing the positioning variations and increasing the repeatabiliy.

### Assesment of the Cast Model Analysis Measurements

Previously, a micrometer device has been used as a control method for measuring enamel thickness.^4,19^ Korkut et al^19^ and Bozkurt et al^4^ used a screw-type digital micrometer directly on extracted teeth. In the present study, the same micrometer was used on cast models as the control method for clinically evaluating enamel thickness on worn incisal edges. As the micrometer was an industrial type digital device, it was impossible to sterilise it for infection control; therefore, it was performed indirectly on cast models to measure incisal tooth wear. Besides, the micrometer served as the control method in this study, so that it was recommendable to have a stable cast as in vitro studies. The readings from the cast model analysis gradually decreased statistically significantly during the overall evaluation period, and the measurements at each period were statistically different for both groups (p < 0.001) (Fig 2). Although the enamel thickness measurements for cast model analysis and ultrasound were different as a result of difference reference points, a positive and significant correlation (p < 0.001) was found between the two techniques regarding the differences in measurements for the overall evaluation period (T1-T5) (Table 2).

### Assessment of the Digital Radiography Measurements

Digital radiography was previously used to assessing the rate of tooth wear in maxillary and mandibular central incisors in a cross-sectional study.^20^ The crown lengths of permanent maxillary and mandibular central incisors were measured based on digital radiographic images. However, the evaluation period of the study of Ray et al^30^ was six decades, therefore significant length decreases were observed in milimeters. In our study, digital radiography could not detect progressive incisal tooth wear under 100 µm clinically, supporting the results of Korkut et al.^19^ The reason for not detecting could be the 100-µm sensitivity limit of the computer software used here to take measurements on radiographic images. Korkut et al^19^ used the same software for detecting incisal tooth wear; however, the amount of artificial abrasion they performed per abrasion cycle was more than 100 µm, thus digital radiography software could detect the changes. According to our results, only the readings of 24 and 48 months were statistically significantly different (p < 0.001) for group 2, showing that incisal tooth wear for the patients who did not use splints was greater than 100 µm for the 2-year period (Table 1).

### Assessment of the Colorimeter Measurements

The colorimeter method has been previously used for short-term evaluation of the light absorbance due to a formed colored complex, reflecting the degree of hard tissue loss from the surface.^16^ Krikken et al^20^ evaluated colorimeter readings for monitoring incisal tooth wear in vitro and concluded that it was impossible to estimate the remaining enamel thickness, but that the colorimeter could be suitable for monitoring progression of erosive enamel loss. In the present study, the colorimeter detected the darkening of incisal color in group 1 from 24 months, but in group 2 from 12 months (Fig 2). As the darker colors in natural teeth tend to increase from incisal to cervical,^11^ the color of the worn surfaces gradually become darker, in accordance with progressive incisal wear. However, because of the limitations in measurement sensitivity of the colorimeter device, color changes equal to a mean of 40-µm hard tissue loss were detected at least from the first year evaluations (for group 2), corresponding to the ultrasound and cast model measurements (Table 1). Supporting the results of Krikken et al,^20^ the colorimeter might have clinically monitored the progression of incisal tooth wear, but with a limitation: a minimum 40-µm hard-tissue loss was necessary to yield detectable and significant darkening in incisal color under the conditions of this study. In order to obtain a more precise result, other factors (GERD, diet, smoking, etc) that darken the color should be evaluated in further studies.^23,27^ A positive, statistically significant correlation was also found between ultrasound and colorimeter for T3, T4, and T5 regarding the differences in evaluation periods (p < 0.001) (Table 2).

### Assessment of the FluoreCam Measurements

The auto-fluorescence of enamel decreases with the decreased mineral content.^16^ Based on this principle, light-induced fluorescence devices were suggested for quantitatively monitoring the mineral changes in vitro and in vivo. However, the clinical effectiveness of the FluoreCam device has been the subject of debate.^5^ Abufarwa et al^1^ reported FluoreCam as a highly reliable and valid device for in vitro assessment of enamel demineralisation. Yanikoglu et al^43^ indicated that FluoreCam could detect enamel caries on flat surfaces in vitro, supported by Gocmen et al^9^ who detected mineral density changes in vitro in enamel demineralisation. In a clinical study, Korkut et al^18^ used FluoreCam to detect demineralisation and remineralisation areas surrounding orthodontic brackets, where it was considered sensitive in clinically measuring weekly alterations in mineral content. Durmus et al^6^ also mentioned FluoreCam as a useful method for clinically monitoring molar-incisor hypomineralisation. Korkut et al^19^ used FluoreCam in vitro to examine progressive incisal tooth wear for the first time. FluoreCam detected the decrease in mineral content in accordance with incisal reduction cycles, which also supported the fact that mineral content decreases from enamel to dentin.^16^ However, they also mentioned greater consistency of the device for measuring inorganic content (enamel tissue) and less consistency with a higher amount of dentin exposure. FluoreCam was produced based on the assumption of 87% inorganic content for effective measuring, which means it was mainly designed to measure enamel rather than dentin. In this study, the teeth with minimal dentin exposure (occlusal/incisal wear severity scale15, grade 2) were included; FluoreCam detected statistically significant progressive mineral loss clinically at each evaluation period for both groups (p < 0.001) (Fig 3), supporting the results of other studies.^1,9,18,19^ Positive, statistically significant correlations were obtained between FluoreCam and ultrasound/cast model analysis measurements, which might indicate the reliability of FluoreCam method (p < 0.001) (Table 2).

### Comparisons between the Groups

In a recent systematic review, splints were mentioned as helpful for managing tooth wear caused by bruxism.^31^ Clinical studies in the literature focused on occlusal splints for patients with bruxism, and generally considered pain instead of tooth wear as the primary outcome.^7,17^ Very few studies have provided usable longitudinal data about the interaction of bruxism and occlusal splints,^7,10,29^ so that we were unable to determine whether splints were effective or not.^31^ No evidence of adverse effects was reported with splints, but there was also no evidence that splints increased mouth opening, reduced TMD clicking, or improved the quality of life.^29,31^ A systematic review and meta-analysis reported moderate-quality evidence that splint therapy had substantial effect on reducing pain, but no significant effect on quality of life or depression.^7^ Furthermore, most studies had a high risk of bias due to the impossibility of blinding patients to wearing a splint or not,^31^ which was also valid for the present study.

In this clinical study, the effect of the night guards (hard occlusal splints) was longitudinally investigated. The mean amount of hard tissue loss on incisal edges gradually increased for both groups during the overall evaluation period. That was probably because of the increasing rate of wear of the hard tissue surfaces in accordance with the increased dentin exposure as well as decreased mineral content.^19^ Regarding group 1 (the patients who used night guards for 6 months), the decrease of the mean crown length for maxillary incisors was 20 µm in 1 year, 43 µm in 2 years, and 103 µm in 4 years. Regarding group 2 (the patients who did not use night guards), the decrease of the mean crown length for maxillary incisors was 43 µm in 1 year, 91 µm in 2 years, and 186 µm in 4 years (Table 3). A previous study reported the decrease of the mean crown length as 1 mm for maxillary incisors and 1.5 mm for mandibular incisors in six decades.^30^ Our results for patients with nocturnal bruxism agreed with the previous results. Accordingly, the statistically significantly lower decrease in the mean crown length for group 1 can be interpreted to mean that the use of night guards for 6 months might be effective in preventing incisal tooth wear among patients with nocturnal bruxism. Both ultrasound and cast model analyses detected the periodical statistically significant decreases for 1, 2, and 4 years between the groups (p < 0.001) (Table 3), but they did not detect statistically significant differences at momentary follow-up measurements (p ≥ 0.05) (Table 1).

In terms of changes in color and mineral content, colorimeter and FluoreCam readings also revealed significant differences between the groups. These differences between the groups for colorimeter readings were determined at 24 months (p = 0.010) and 48 months (p < 0.001). The statistically significant decreases in mean crown length for group 2 (Table 3) corresponded to significant darkening in color (Table 1). Therefore, gradual darkening of color might have indicated progressive tooth wear on the insical surfaces. Our finding of greater color darkening in group 2 than in group 1 at two years could be mean that splint use might have provided muscle relaxation and/or a habit-breaking effect,^10^ so that the level of hard tissue loss from the surface was lower in the patients who wore splints. However, other factors such as GERD, diet, and smoking should be considered, which might also have influenced discoloration.^23,27^

Similar to the colorimeter results, the FluoreCam of were also statistically significantly different between the groups at 12, 24, and 48 months, revealing more mineral loss for group 2 (p < 0.001) (Fig 3). The statistically significant decreases in mean crown length in group 2 also corresponded to statistically significant decreases in mineral content (Table 1). This result could indicate that more hard tissue might be worn away in the patients who did not wear splints, especially at 12 months and the subsequent evaluation periods. The compatible results of colorimeter and FluoreCam could indicate that the level of wear might be higher in the patients who did not use night guards, supporting the ultrasound and cast model results that the use of a night guard might have inhibited the decrease in mean crown length.

## Conclusion

This clinical follow-up study particularly focused on tooth wear as the primary outcome regarding the evaluation of effectiveness of occlusal splints for bruxism. The clinical progression of incisal tooth wear was monitored quantitatively. The precision of the measurements varied between the diagnostic methods used.

The decrease in the mean crown length in group 1 was significantly lower than in group 2 1- (p<0.001), 2- (p<0.001), and 4-year (p<0.001) follow-ups. Thus, within the limitations of this study, it may be concluded that the use of night guards for 6 months might be effective in preventing the incisal tooth wear in patients with nocturnal bruxism. Ultrasound was found to be a quantitative, reliable, and repeatable method for clinically monitoring progressive incisal tooth wear <10 µm. FluoreCam could clinically monitor the mineral content changes on worn incisal surfaces in accordance with progressive tooth wear. The colorimeter could clinically monitor darkening of incisal color with a hard-tissue-loss limitation of 40 µm or greater. Digital radiography could not clinically detect progressive incisal tooth wear. FluoreCam and colorimeter records might have revealed the potential protective effect of occlusal splints for progressive tooth wear.
